# Supplementary material for: Interventions for Shiga toxin-producing Escherichia coli gastroenteritis and risk of hemolytic uremic syndrome: A population-based matched case control study
Source: PLoS One. 2022 Feb 4;17(2):e0263349. doi: 10.1371/journal.pone.0263349 (PMC8815883; doi:10.1371/journal.pone.0263349)
Supplement: S1 File — (DOCX) [file pone.0263349.s001.docx]

# **Supporting information**

**S1 Table. Power analysis of 100 cases and 500 controls to detect odds ratios with clinical significance**

|  | Exposure to antimicrobial agents in the non-HUS group (%) | | |
| --- | --- | --- | --- |
| Odds ratio in the alternative hypothesis | 75 | 80 | 85 |
| 0.375 | 99.0% | 98.3% | 96.0% |
| 0.400 | 97.9% | 96.4% | 92.6% |
| 0.425 | 95.8% | 93.4% | 87.9% |

HUS, hemolytic uremic syndrome

**S2 Table. Univariable and multivariable conditional logistic regression analysis to evaluate the association between Fosfomycin administration and development of HUS only in patients detected with serotype O157**

|  |  |  | **Matched OR (95% CI)** | | **Matched OR (95% CI)** | |
| --- | --- | --- | --- | --- | --- | --- |
|  | **Cases** | **Controls** | **Unadjusted** | ***P* value** | **Adjusted** | ***P* value** |
| All ages | 26/65 | 114/208 | 0.58 (0.34-0.98) | 0.04 | 0.68 (0.40-1.15) | 0.147 |
| Children | 19/47 | 95/133 | 0.42 (0.23-0.76) | 0.004 | 0.52 (0.28-0.97) | 0.041 |
| Adults | 7/18 | 19/75 | 1.53 (0.59-3.95) | 0.384 | 1.28 (0.48-3.45) | 0.623 |

Unadjusted matched odds ratios were calculated by univariable conditional logistic regression analysis. Adjusted matched odds ratios were calculated by multivariable conditional logistic regression analysis using the following covariates: age, sex, area, presence of bloody stool, initial white blood cell count, initial C-reactive protein level, and use of antidiarrhoeal agents.

CI, confidence interval; HUS, hemolytic uremic syndrome; OR, odds ratio

**S3 Table. Univariable and multivariable conditional logistic regression analysis to evaluate the association between the Shiga toxin-producing *Escherichia coli* strain O157 and development of HUS**

|  |  |  | **Matched OR (95% CI)** |  | **Matched OR (95% CI)** |  |
| --- | --- | --- | --- | --- | --- | --- |
|  | **Cases** | **Controls** | **Unadjusted** | ***P* value** | **Adjusted** | ***P* value** |
| All ages | 65/90 | 216/371 | 1.83 (1.11–3.00) | 0.017 | 0.71 (0.41–1.23) | 0.224 |
| Children | 47/68 | 140/266 | 1.84 (1.07–3.16) | 0.027 | 0.70 (0.38–1.29) | 0.254 |
| Adults | 9/22 | 24/104 | 1.75 (0.51–5.99) | 0.374 | 0.43 (0.07–2.49) | 0.345 |

Unadjusted matched odds ratios were calculated by univariable conditional logistic regression analysis. Adjusted matched odds ratios were calculated by multivariable conditional logistic regression analysis using the following covariates: age, sex, area, presence of bloody stool, initial white blood cell count, initial C-reactive protein level, use of any antibiotics, and use of antidiarrheal agents.

CI, confidence interval; HUS, hemolytic uremic syndrome; OR, odds ratio

**S4 Table. Univariable and multivariable conditional logistic regression analysis to assess the effect of the timing of fosfomycin use within five days of illness on HUS development.**

|  |  |  | **Matched OR (95% CI)** | | **Matched OR (95% CI)** | |
| --- | --- | --- | --- | --- | --- | --- |
|  | **Cases** | **Controls** | **Unadjusted** | ***P* value** | **Adjusted** | ***P* value** |
| All ages | 32/90 | 147/371 | 0.76 (0.48-1.19) | 0.227 | 0.85 (0.53-1.36) | 0.499 |
| Children | 24/68 | 125/266 | 0.67 (0.41-1.11) | 0.124 | 0.77 (0.45-1.29) | 0.315 |
| Adults | 6/22 | 20/105 | 1.23 (0.47-3.20) | 0.673 | 1.19 (0.38-3.76) | 0.764 |

Unadjusted matched odds ratios were calculated by univariable conditional logistic regression analysis. Adjusted matched odds ratios were calculated by multivariable conditional logistic regression analysis using the following covariates: age, sex, area, presence of bloody stool, initial white blood cell count, initial C-reactive protein level.

CI, confidence interval; HUS, hemolytic uremic syndrome; OR, odds ratio
